# The Yellow Fever Virus Type-Specific Epitope Recognized by Monoclonal Antibody 2D12 Neutralizes Wild Type, but Not Live Attenuated 17D or French Neurotropic Vaccine Strains

**DOI:** 10.3390/vaccines14050430

**Published:** 2026-05-12

**Authors:** Clairissa A. Hansen, Shawn Rast, Jill K. Thompson, Haiping Hao, Daniel Jupiter, Stephen Higgs, Nigel Bourne, Alan D. T. Barrett

**Affiliations:** 1 Department of Pathology, University of Texas Medical Branch (UTMB), Galveston, TX 77555, USA; 2 Sealy Institute for Vaccine Sciences, University of Texas Medical Branch (UTMB), Galveston, TX 77555, USA; 3 Independent Researcher, Austin, TX 78728, USA; 4 Department of Biochemistry and Molecular Biology, University of Texas Medical Branch (UTMB), Galveston, TX 77555, USA; 5 Department of Biostatistics and Data Science, University of Texas Medical Branch (UTMB), Galveston, TX 77555, USA; 6 Department of Orthopedic Surgery and Rehabilitation, University of Texas Medical Branch (UTMB), Galveston, TX 77555, USA; 7 Biosecurity Research Institute, Kansas State University, Manhattan, KS 66502, USA; 8 Department of Diagnostic Medicine/Pathobiology, College of Veterinary Medicine, Kansas State University, Manhattan, KS 66502, USA; 9 Department of Pediatrics, University of Texas Medical Branch (UTMB), Galveston, TX 77555, USA

**Keywords:** live attenuated vaccine, yellow fever, monoclonal antibody epitope, virus neutralization

## Abstract

**Background/Objectives**: The envelope (E) protein of orthoflaviviruses contains antigenic sites that are composed of one or more epitopes, which can vary in antigenic specificity, including between viral species, strains, and even substrains. Monoclonal antibodies (mAbs) that bind these epitopes vary in functionality based on their specificity. This makes mAbs useful to study the differences in phenotypes between strains of viruses, such as the wild type (WT) and live attenuated vaccine strains of yellow fever virus (YFV). mAb 2D12 was raised against the 17D-204 YFV vaccine substrain virus (YF VAX^®^) by Schlesinger et al. in 1983. However, it only neutralizes Asibi WT virus, not the 17D-204 vaccine substrain virus. **Results**: We confirmed these results and demonstrated that mAb 2D12 fails to neutralize all 17D vaccine substrains (17D-204, 17DD, and 17D-213), indicating that the minor differences between these virus substrains do not affect the epitope or functionality of mAb 2D12. In addition, mAb 2D12 was found to neutralize WT strain of French viscerotropic virus (FVV), with statistically indistinguishable neutralization from the WT strain Asibi. All but one of the live attenuated French neurotropic vaccine (FNV) derivative viruses had significantly lower neutralization than WT strains Asibi and FVV. FVV, Asibi, 17D, and FNV have many amino acid differences in the membrane (M) and E proteins. It is unclear which of them contributes to this differential neutralization. However, FNV and 17D have common amino acid substitutions from WT FVV and Asibi at positions M-36 and E-331, suggesting that one or both of these residues may contribute to the 2D12 epitope. **Conclusions**: Overall, mAb 2D12 is a valuable tool to distinguish WT virulent strains of YFV from live attenuated vaccine strains.

## 1. Introduction

The yellow fever virus (YFV) is the prototypical member of genus *Orthoflavivirus*. It is an arthropod-borne virus (or arbovirus) primarily spread between primates and mosquitoes of the *Aedes*, *Sabethes*, and *Haemagogus* genera.

The flavivirus genome is a single-stranded, positive-sense RNA that is approximately 11 kilobases long. The genome consists of a 5′ untranslated region (UTR), a single open reading frame that encodes a polyprotein, and a 3′ UTR. The polyprotein is co- and post-translationally cleaved into three structural proteins (capsid (C), pre-membrane/membrane (prM/M), and envelope (E)) and seven nonstructural proteins (NS1-5). The structural proteins, together with the genomic RNA, assemble into virions that are approximately 50 nm in diameter. The E protein is responsible for binding the virus to cell receptors and fusion inside the host cell. It comprises much of the surface area of the virion, is the primary immunogen for YFV, and has many epitopes that elicit neutralizing antibodies [1,2,3,4]. The prM protein acts as an E protein chaperone during viral replication and assembly. It is cleaved to become an M as the virus matures while exiting the host cell. Although there are epitopes on M and prM proteins, they often elicit poor neutralization activity, as they are hidden and less available on the surface of the virion. The C protein forms the nucleocapsid and binds the viral genomic RNA inside the virion. The NS proteins form the replication complex and are involved in replication and inhibition of the host innate immune response, among other functions.

There are no specific antiviral treatments for yellow fever (YF), but the disease is vaccine-preventable, and the primary method for controlling the disease is widespread vaccination. The live attenuated vaccine, called 17D, was developed in the late 1930s by Max Theiler and coworkers [5,6], and was generated by passaging the wild-type (WT) strain Asibi 176 times in mouse and chick embryo tissue. In addition to the loss of viscerotropism, neurotropism, and vector infectivity, immunity from one dose of the 17D vaccine lasts at least 10 years, if not for life [7]. The molecular basis of attenuation and strong immunogenicity are poorly understood because it is a legacy vaccine that was derived empirically, rather than by rational design. However, studies have shown that neutralizing antibodies are the correlate of protection [7].

In addition to the 17D vaccine, the French neurotropic vaccine (FNV) was derived following 128 passages in mouse brain of the WT parental strain, the French viscerotropic virus (FVV) [8,9,10]. Like the 17D virus, FNV had a loss of viscerotropism and vector infectivity. Unlike 17D, FNV had enhanced neurotropism and is no longer in use today.

The 17D vaccine exists as three substrains: 17D-204, 17D-213, and 17DD. There are 20 common amino acid substitutions between all the 17D substrains and the WT parent strain Asibi, including [9] one in the E protein and one in the M protein (Supplemental Table S1). The three 17D substrain viruses differ at 14 amino acid positions in the polyprotein, with five being in the E protein (Supplemental Table S2). FNV also has 20 amino acid substitutions from WT FVV, including two in C, one in prM, one in M, and four in the E protein; However, these are in different amino acid positions than those that distinguish Asibi from 17D, except for two of these substitutions (M-36 and E-331) (Supplemental Table S1) [11].

There have been multiple studies comparing the Asibi and 17D viruses with E protein-reactive monoclonal antibodies (mAbs), utilizing methods including physical binding, competition binding assays, hemagglutination inhibition, neutralization, and passive protection in mouse models [1,4]. These studies identified a variety of epitopes on the E protein with different orthoflavivirus specificities, including epitopes that are 17D-204 substrain-specific, 17DD substrain-specific, 17D and FNV-specific, WT-specific, YFV type-specific, and those common to multiple orthoflaviviruses.

Schlesinger et al. in 1983 tested a panel of mouse mAbs for neutralization against the Asibi and 17D-204 viruses using a log10 neutralization index (LNI) [1]. In this assay, the antibody quantity/concentration is held constant, while the infectivity titer of the virus is varied in the presence and absence of the mAb using ten-fold dilutions of the virus. Then, the fold-change in titer is calculated between these two values and log_10_-transformed to obtain the LNI value. An LNI of 0 indicates an undetectable difference in titer after neutralization and no neutralizing activity against the virus strain, whereas an LNI of three indicates a 1000-fold difference in the infectivity titer and high levels of neutralization against the virus strain. In comparison, most neutralization assays today use a constant amount of virus and varying dilutions of antibody, e.g., the Plaque Reduction Neutralization Test (PRNT). Schlesinger and colleagues used the LNI to measure neutralization because historical studies showed that non-human primates (NHPs) are to be protected, as they produce enough neutralizing antibodies against YFV to confer an LNI of 0.7 [12]. This is also the value that determines the cutoff of seropositivity in some clinical trials [13]. Therefore, to maintain relatively standard methodologies between studies, in this paper, the LNI with constant antibody concentration was utilized rather than the varied antibody concentration, PRNT. Also, it was not necessary to determine the exact amount of antibody required to neutralize the virus (as in a PRNT); instead, the goal was simply to establish whether the constant quantity of antibody could neutralize the virus at all.

In the Schlesinger et al. studies, the panel of mAbs was split into groups A-E based on a combination of physical and biological properties. Schlesinger et al. designated groups B and C as containing mAbs that were YFV type-specific. mAbs in group B (4E8, 2C9, and 2E10) neutralized both WT Asibi and 17D-204 vaccine viruses, whilst mAbs in group C (2B8, 5E3, 2D12, 3A3, and 4E1) neutralized Asibi virus, but not 17D-204 vaccine virus in an LNI assay [1,4]. In particular, mAb 2D12 neutralized Asibi virus, with an LNI of 4.3, but only neutralized 17D-204 virus with an LNI of <1.3, even though this antibody was generated by immunizing mice with 17D-204 virus [1]. In the original studies, only 17D-204 was tested, not the other two 17D substrains, nor FNV or FVV.

In this study, we sought to confirm and extend the studies of Schlesinger et al. Specifically, we aimed to investigate the ability of mAb 2D12 to neutralize the two other 17D substrains (17DD and 17D-213), plus FNV and FVV viruses, to determine whether all vaccine strains of YFV can escape neutralization by mAb 2D12 or if this biological activity is specific only to the 17D-204 vaccine strain. The findings from this study could lead to insights into the antigenic differences between the safe, immunogenic YFV vaccine strains and the pathogenic WT strains.

## 2. Materials and Methods

### 2.1. Recovery of YFV Infectious Clone (ic)

The YFV ic is a single plasmid containing the full genome of either the Asibi or 17D-204 strain [14,15]. *E. coli* XL10 gold cells from Agilent (Cedar Creek, TX, USA) were transformed with plasmids and grown with 100 µg/mL of ampicillin on Luria broth (LB) 2% agar plates. Single colonies were picked and grown overnight (14–16 h) in 200 mL LB with ampicillin in a shaker at 37 °C, 225 rpm. In total, 1 mL of bacterial stock was mixed with 50% glycerol and stored at −80 °C as a DNA stock. The bacterial cells were pelleted and resuspended in glucose-Tris-EDTA (GTE). They were then lysed with 0.2 M NaOH/1% SDS. The lysis was neutralized with 2 M KaOH, and the DNA was precipitated with isopropanol. Phenol:chloroform:isoamyl alcohol and chloroform were used to purify the DNA. It was then purified using the Qiagen (Venlo, The Netherlands) QIAquick PCR Purification Kit according to the manufacturer’s protocols.

To linearize the YFV genomes and excise them from the rest of the plasmid, 4 µg of the plasmid was digested for 2 h at 37 °C using *Nru1* and *Xho1* (NEB, Ipswich, MA, USA) for the Asibi ic and 17D ic, respectively. The proteins were digested with proteinase K (NEB) for one hour at 37 °C, and the DNA was purified again with phenol:chloroform and ethanol/sodium acetate precipitation. The linear DNA genomes were eluted in 10 µL of DNAse-free RNAse-free water. The DNA was transcribed into RNA using the CellScript (Madison, WI, USA) Amplicap SP6 High Yield Message Maker kit according to the manufacturer’s protocol. The RNA was added to either 6.8 × 10^6^ Vero or C6/36 cells in 0.5 mL of Dulbecco’s phosphate-buffered saline (DPBS). The cells were electroporated with 1.5 kV, 25 µF, and ∞ ohms using a Gene Pulser (Bio-Rad, Hercules, CA, USA) and incubated at room temperature (RT) for 10 min. Cells were grown as stated below until a cytopathic effect was observed, upon which the viruses were collected, aliquoted, and stored at −80 °C.

### 2.2. Virus Stocks

All viruses used are listed in Supplemental Tables S3–S5. All viruses were stored at −80 °C until use. The viruses and strains used in these studies have been described previously [11,16,17]. Most virus stocks used had infectivity titers greater than 5 log_10_ ffu/mL to ensure accurate neutralization data.

### 2.3. Cell Culture

Vero monkey kidney (ATCC (Manassas, VA, USA) CCL-81) cells were grown with 5% CO_2_ at 37 °C in 1× minimum essential media (MEM), 8% fetal bovine serum (FBS), 100 units/mL penicillin, 100 µg/mL streptomycin, 0.1 mM non-essential amino acids (NEAAs), and 2 mM L-glutamine. When cells were infected with a virus, the same media was utilized except with 2% FBS.

### 2.4. Virus Titrations

#### Focus-Forming Assays (FFAs)

A focus-forming assay (FFA) was used to calculate all viral titers. Vero cells were seeded to 80–90% confluency. Viruses were serially diluted in a 10-fold series. After washing with DPBS, 100 µL of each dilution of virus was added to individual wells. The plates were rocked for 30 min at room temperature. The infected cells were overlayed with 1 mL MEM with 2% FBS and 0.8% carboxymethyl cellulose (CMC) for each well. The plates were incubated for 4 days at 37 °C with 5% CO_2_. The CMC was removed from the cells, and the cells were fixed with 1:1 acetone:methanol. After at least 30 min of exposure to acetone:methanol, the solution was removed and the plates were allowed to dry. An amount of 1 mL of blocking buffer (PBS supplemented with 1% FBS) was added to each well and rocked at RT for 30 min. The block was then removed, and the plates were washed thoroughly with PBS. Anti-Asibi mouse immune ascites fluid (MIAF) (World Reference Center for Emerging Viruses and Arboviruses: WRCEVA, Galveston, TX, USA) was added to each well in 100 µL volume diluted to 1:5000. After at least one hour, the wells were washed again with PBS. An amount of 100 µL of the secondary antibody goat anti-mouse IgG with biotin was added at a 1:500 dilution and incubated for at least one hour at RT. Cells were again washed with PBS, and 100 µL of neutravidin-HRP at a 1:3000 dilution was added to each well and incubated for at least one hour at RT. Plates were washed with PBS, and the foci of infected cells were developed with 3,3′-diaminobenzidine (DAB) according to the manufacturer’s instructions for 5–20 min. The reaction was quenched with water, and the foci were counted. The titer was determined using the reciprocal dilution of the most dilute well containing over five foci. Titrations were recorded in focus-forming units (FFU)/mL.

### 2.5. Log_10_ Neutralization Index (LNI)

In total, 12 µL of MAB984 (Anti-Yellow Fever Virus Antibody, clone 2D12.A, ascites, EMD Millipore (St. Louis, MO, USA), neutralization (Asibi YF: 1:1000–1:3000)) or 12 µL of PBS as a control were mixed with 108 µL of YFV stocks, undiluted. Solutions were pulse-vortexed once to ensure a homogenous mixture. Solutions were incubated in a 37 °C water bath for exactly 30 min. Viruses were titrated using the FFA. The fold-change in infectivity titer between the control virus (YFV mixed with PBS) to that of the mAb 2D12-neutralized virus (YFV mixed with a constant quantity of mAb 2D12) was calculated (control infectivity titer divided by infectivity titer following neutralization by mAb 2D12) and log_10_-transformed to get the final LNI value.

### 2.6. Next-Generation Sequencing (NGS) Pipeline

Viral RNA was extracted from the undiluted virus stocks utilizing the Qiagen Viral RNA Mini Kit according to the manufacturer’s instructions. Samples were transported to the UTMB Next-Generation Sequencing Core on dry ice. cDNA libraries were prepared with random hexamer priming and sequenced on the Illumina (San Diego, CA, USA) NextSeq 550 instrument for paired-end 75 bp reads. The raw reads were trimmed using Trimmomatic (v 0.39) with a cutoff set at Q30. Reads shorter than 35 bases were discarded. The reads were aligned to published genomes of Asibi, 17D-204, FVV, and FNV viruses, and were aligned using Bowtie2 software (v 2.5.3) with paired-end, very sensitive local parameters. Picardtools MarkDuplicates was used to remove PCR duplicates (Picardtools (v 3.1.1), Broad Institute, MIT). Each sample was down-sampled to match the sample with the lowest mean coverage above 1000 (1091 for this study). Single-nucleotide variants (SNVs) were identified with the lofreq (Genome Institute of Singapore, v 2.1.0) program, and all SNVs that had greater than 1% frequency were noted. Multiple papers have been published that include deep sequencing of yellow fever virus strains and have used 1% SNV frequency as a statistical cutoff to ensure that artifacts or low-quantity variants are less likely to be included in the analysis (References [11,18]). All programs and scripts noted above were streamlined into a one-command pipeline by Shawn Rast. Shannon entropy was calculated in R(v 4.2.1) using custom scripts provided by Kassandra Carpio, PhD (UTMB), using a formula published by Nishijima et al. [19].

The data for full viral genome sequences have been deposited under the NCBI GenBank accession numbers: PX648516, PX660172-PX660175, and PX766920-PX766933. Next-generation sequencing data are available through NCBI’s Sequence Read Archive (SRA) under BioProject PRJNA1401925. The SRA accession numbers are SRR36990668-SRR36990686.

### 2.7. Statistics

All statistical analyses were completed using GraphPad Prism 8 software (Boston, MA, USA). Normally distributed data were assessed for significance using ordinary one-way ANOVA with Dunnett’s multiple comparisons test to compare all samples to one baseline sample. Data that were not normally distributed were assessed for significance using the Brown–Forsythe and Welch ANOVA tests with Dunnett’s multiple comparisons test. Welch’s corrected *t*-test was used when only two samples were being compared. Samples with adjusted *p*-values below 0.05 were considered significant.

## 3. Results

### 3.1. WT Asibi Virus Is Neutralized by mAb 2D12 While 17D Vaccine Is Not

Despite being well-established virologic procedures for many years, LNI and PRNT neutralization assays for YFV have not been standardized between laboratories. Therefore, to produce results that would be directly comparable to those described by Schlesinger et al. [1,4], we used the LNI assay rather than the PRNT. The fold-change in the infectivity titer after incubation with mAb 2D12 was calculated and log_10_-transformed to produce the final LNI results. After incubation with mAb 2D12, Asibi virus had an LNI of 3.45 (standard error ±0.19, n = 25), and 17D-204 virus had an LNI of 0.05 (standard error ±0.06, n = 31) (Table 1). These results were statistically significant (Welch’s corrected *t*-test; *p* < 0.0001), and the LNI results were comparable to the original studies of Schlesinger et al., which were 4.3 and <1.3, respectively [1]. The difference in results for Asibi between Schlesinger (LNI of 4.3) and this study (LNI of 3.43) may be due to the quantity of mAb 2D12 in the ascitic fluids used by Schlesinger et al. [1,4] and the mAb-containing ascitic fluid provided commercially by EMD Millipore for the current study. Overall, we confirm the results of Schlesinger et al. [1] that describe the high neutralization of Asibi virus and low-to-undetectable neutralization of 17D vaccine by mAb 2D12.

### 3.2. Infectious Clones vs. Isolated Viruses

Viruses that have been isolated from vaccine stocks or live hosts may better represent what the viruses do in the “wild”, but they also have the caveat of undergoing passages in different cell cultures and hosts, which potentially introduces more genetic variability in the viral RNA population. This includes genetic variability between stocks of the same virus that have been shown to have the same sequence but different passage history. Infectious clones, on the other hand, tend to have lower genetic heterogeneity due to their low passage history and derivation from cDNA. This makes them useful tools to specifically study the amino acid differences between viruses.

**Figure 1:**
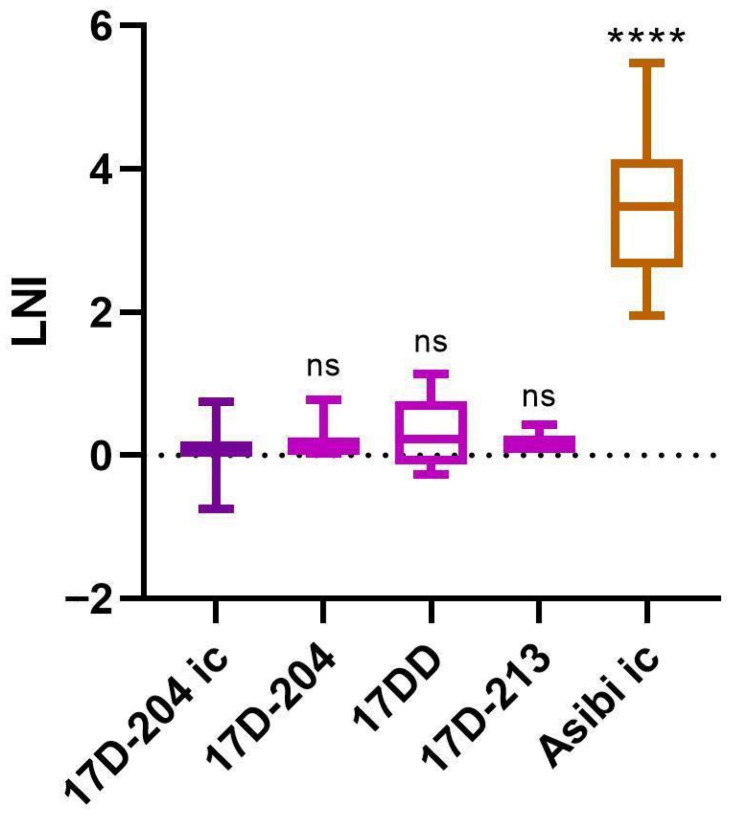
LNI results for the 17D substrains. Welch’s one-way ANOVA for unequal variances and Dunnett’s multiple comparison tests were utilized for statistics. All samples were compared to the control sample 17D-204 ic. ns: not significant; **** *p*-value < 0.0001.

**Figure 2:**
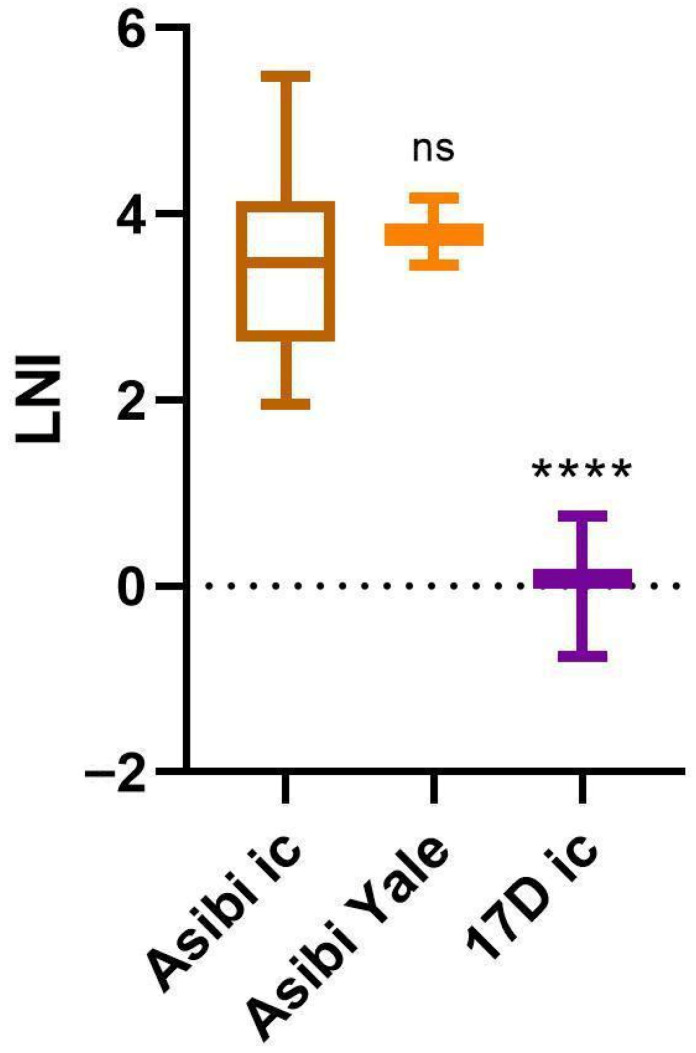
LNI results for the Asibi non-ic-derived virus. Welch’s one-way ANOVA for unequal variances and Dunnett’s multiple comparison tests were utilized for statistics. All samples were compared to the control sample Asibi ic. ns: not significant; **** *p*-value < 0.0001.

The LNI after exposure of 17D-204 YF-VAX^®^ to mAb 2D12 did not differ significantly from that of its ic counterpart (Figure 1 and Supplemental Table S3), i.e., 0.31 vs. 0.05, respectively. The same was true for the LNI of the non-ic Asibi Yale virus and that of its ic counterpart (Figure 2 and Supplemental Table S4), i.e., 3.80 (n = 3, standard error = 0.21) vs. 3.45 (n = 25, standard error = 0.19), respectively. Therefore, the ic-derived viruses can be reliably used for these assays.

### 3.3. 17D Vaccine Substrains Are Not Neutralized by mAb 2D12

The LNI values for the 17D substrain viruses (17D-204 YF-VAX^®^, 17DD Brazil Bio-Manguinhos/FIOCRUZ, and 17D-213 Russia Chumakov Institute) did not differ significantly when compared to 17D-204 ic (Figure 1 and Supplemental Table S3) [LNI of 0.31 (n = 3; standard error = 0.24), 0.31 (n = 6; standard error = 0.21), 0.24 (n = 3; standard error = 0.09), and 0.05 (standard error ±0.06, n = 31), respectively]. The LNI values for the non-IC 17D viruses are slightly higher than those of the ic-derived virus. Overall, these data indicate that all three 17D substrains are not neutralized by mAb 2D12.

### 3.4. 17D Vaccine and FNV Strains Are Neutralized Less than WT Strains Asibi and FVV by mAb 2D12

**Figure 3:**
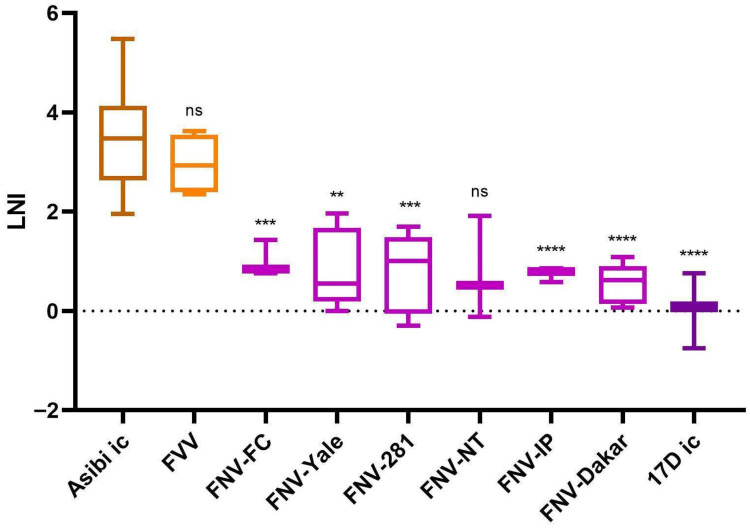
LNI results for the FNV strains after exposure to mAb 2D12. Welch’s one-way ANOVA for unequal variances and Dunnett’s multiple comparison tests were utilized for statistics. All samples were compared to the control sample Asibi ic. ns: not significant; ** *p*-value < 0.01; *** *p*-value < 0.001; **** *p*-value < 0.0001.

This study utilized six distinct examples of FNV from varying sources [11]. Their amino acid differences can be found in Supplemental Table S1. In neutralization assays with mAb 2D12, five of the six FNV examples (all but FNV-NT) had statistically significantly lower LNI values than Asibi ic (3.45) (Figure 3 and Supplemental Table S5). In order from highest to lowest, the LNI values were FNV-FC: 1.01 (n: 3; standard error: 0.21; *p*-value: 0.0009), FNV-Yale: 0.85 (n: 5; standard error: 0.36; *p*-value: 0.0043), FNV-281: 0.81 (n: 6; standard error: 0.33; *p*-value: 0.0005), FNV-NT: 0.77 (n: 3; standard error: 0.60; *p*-value: 0.17 ns), FNV-IP: 0.74 (n: 3; standard error: 0.08; *p*-value < 0.0001), and FNV-Dakar: 0.54 (n: 5; standard error: 0.18; *p*-value < 0.0001) (Figure 3 and Supplemental Table S5). FNV-NT had a lower LNI than FNV-281, but its standard error was higher, and therefore its LNI was not statistically different from Asibi virus.

In comparison to 17D ic (LNI: 0.05), only one vaccine strain virus had a statistically different LNI: FNV-IP with an LNI of 0.74 (Supplemental Table S5). Again, while other FNV viruses had higher LNI values, their larger standard errors determined that these values were not statistically different from the 17D virus.

WT FVV (LNI: 2.96; n: 4; *p*-value: 0.77) did not have a significantly different LNI when compared to Asibi virus (Figure 3 and Supplemental Table S5), indicating that it is highly neutralized by mAb 2D12. When the one-way ANOVA and Dunnett’s multiple comparison tests were run with FVV as the baseline comparator, 17D ic and all but one of the FNV isolates were significantly different from the LNI of FVV. The LNI for Asibi virus was still not significantly different from FVV in this case (Supplemental Table S5).

Overall, these results show that when Asibi, FVV, 17D, and FNV are all compared to each other, WT strains Asibi and FVV are very highly neutralized by mAb 2D12, there is no significant neutralization of the three 17D substrain vaccine viruses, and FNV isolates fall between these two neutralization extremes (although the FNV isolates are closer in LNI to 17D than to Asibi). Thus, these results show that both the 17D and FNV strains of YFV are neutralized far less (at least 100-fold less) by mAb 2D12 than the WT parental strains.

### 3.5. Amino Acid Comparisons

There are several amino acid differences in the prM and E proteins between Asibi, 17D, FVV, and FNV viruses (Supplemental Table S1). Therefore, it is difficult to disentangle the LNI results to identify the mAb 2D12 epitope based on the amino acid differences. One amino acid residue of interest, however, is at M-36. In both WT strains (FVV and Asibi), M-36 is a leucine (L), while in both 17D and FNV strains, M-36 is a phenylalanine (F). Similarly, all FNV and 17D strains used in these studies have an arginine (R) at E-331, while Asibi ic and FVV have a lysine (K). Thus, M-36 and/or E-331 could be key residues in the functionality of mAb 2D12 neutralization (either as contributors to the epitope or as modifiers of the underlying structure of the proteins) of YFV and could therefore contribute to the neutralization difference between the vaccine and WT strains.

### 3.6. Genetic Diversity Does Not Affect LNI

Another possible explanation for the differences in neutralization by mAb 2D12 is that the viral RNA population could have varying levels of genetic diversity, which could affect overall mAb binding and neutralization. To determine whether or not the viral population genetic diversity at a snapshot in time affected the LNI values, NGS was used to calculate and compare the Shannon entropy and SNV percentages of the viruses. FVV had the closest LNI value to Asibi virus (2.96 vs. 3.45, respectively), but its Shannon entropy was significantly lower than that of Asibi and was even lower than that of 17D (Supplemental Table S6). The FNV isolates had average Shannon entropies that ranged from 0.00282 to 0.00555, whereas the Shannon entropy of Asibi was 0.00369. Statistical analysis of these figures showed there was no distinct correlation between Shannon entropy and LNI (Supplemental Table S6). These results are consistent with previous studies [11].

Asibi virus had an average of 6.5 SNVs above 1%, 1.25 above 5%, and 0.50 above 10%, whereas 17D virus had an average of 3.09 SNVs above 1%, 0.27 above 5%, and 0 above 10% (Supplemental Table S7). Only one isolate of FVV was sequenced by NGS, and it had six SNVs above 1%, four above 5%, and four above 10% (Supplemental Table S7). The FNV examples had far more SNVs than any of the other viruses in all percentage categories. For SNVs above 10%, the FNV isolates ranged from 2.25 to 18.5 SNVs on average (Supplemental Table S7). The highest number of SNVs occurred in FNV-281, which had an average of 79.33 SNVs above 1% (Supplemental Table S7). Due to the great amount of variability seen in the SNVs, no pattern was determined to be present between the SNVs and LNI values. Therefore, it was concluded that viral population genetic diversity did not have an effect on the LNI values for these viruses following neutralization by mAb 2D12.

### 3.7. Control Infectivity Titer Does Not Affect LNI

**Figure 4:**
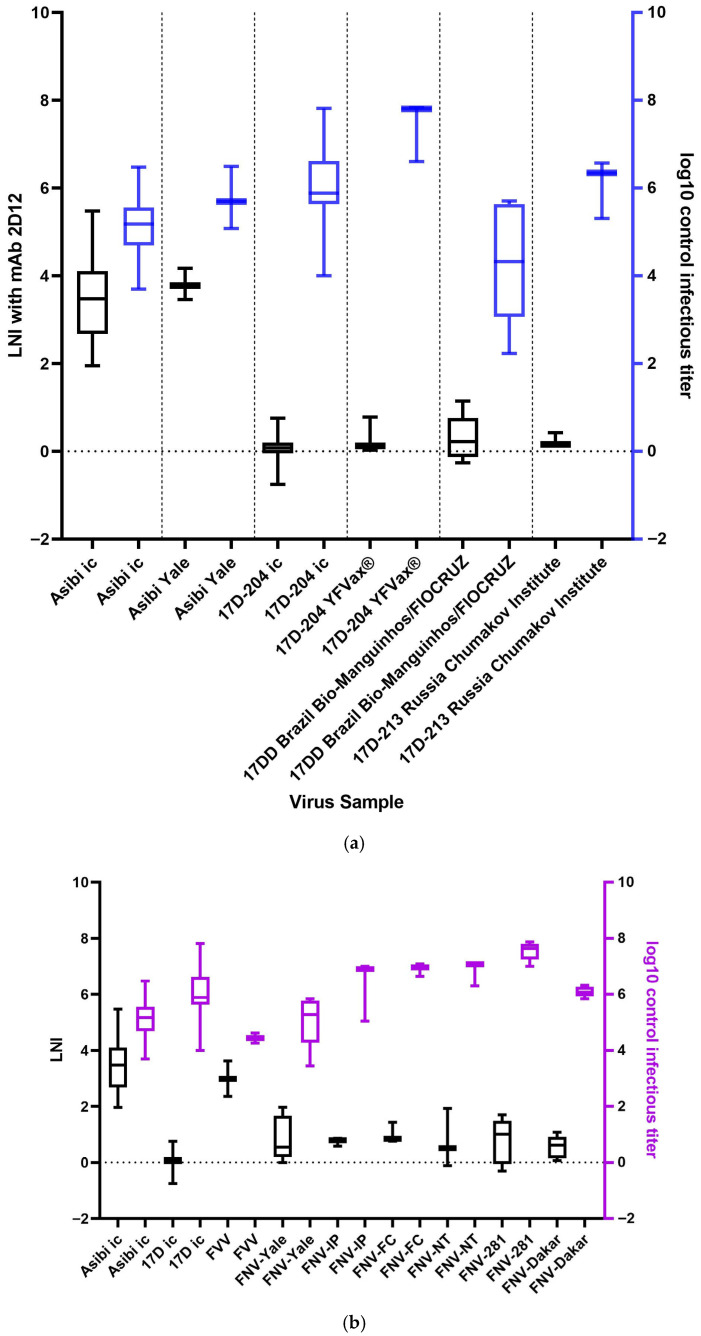
(**a**) LNI values of Asibi and 17D viruses after exposure to mAb 2D12 (left boxplots; black) and log_10_ control titers of those same viruses prior to neutralization (right boxplots; blue). (**b**) LNI values of Asibi, 17D, FNV, and FVV after exposure to mAb 2D12 (left boxplots; black) and log_10_ control titers of those same viruses prior to neutralization (right boxplots; purple).

To determine if altering the control infectivity titer affected the LNI results, the control titers for all viruses tested were compared to one another. Some samples that were tested had lower infectivity titers but were required for use because they could not be grown to higher titers. Infectivity titers for Asibi virus ranged from 2.60 log_10_ ffu/mL to 6.48 log_10_ ffu/mL, while infectivity titers for 17D virus ranged from 4.00 log_10_ ffu/mL to 7.81 log_10_ ffu/mL (Figure 4a). The viruses had varying ranges of infectivity titers, some higher and wider than others. However, there was no discernible pattern between the LNI and control infectivity titer. The LNI values for all FNV isolates were relatively consistent with each other (Figure 4b). As the control titers changed, the LNI did not, as can be seen when FNV-Yale and FNV-281 are compared, for example. No discernible pattern was seen between the control infectivity titer and LNI values. Therefore, the infectivity titer did not appear to influence the LNI.

## 4. Discussion

To this day, LNI and PRNT neutralization assays have not been standardized between laboratories. Therefore, it was important to verify the results of Schlesinger et al. using the LNI [1,4]. Our results confirmed the findings of Schlesinger et al. (1983) [1] that the Asibi virus WT strain was highly neutralized by mAb 2D12, while the 17D vaccine strain was not. Further, the 17D results in this report give a much finer value when compared to the Schlesinger et al.’s original study (0.05 vs. <1.3), which was limited by the laboratory techniques of the time. This study confirms that there is no significant neutralization of the 17D virus by mAb 2D12 (LNI ≤ 0.31) (i.e., the highest LNI of any 17D virus tested).

The 17D-213 and 17DD viruses had similar LNI values to 17D-204, indicating that the small number of amino acid substitutions between these vaccine viruses (including those that impact glycosylation sites) are not involved in the difference in neutralization by mAb 2D12 of 17D and Asibi viruses. Similarly, since the samples of Asibi and 17D viruses derived from ics had the same LNI results as their non-ic counterparts, it is concluded that the ic-derived viruses give reliable results in these assays and can be utilized for further study with Asibi and 17D chimeric viruses in the future. There are 20 common amino acid substitutions between 17D and Asibi viruses: nine are in the structural proteins, including eight in the E protein and one in the M protein. It is likely that one or more of these amino acid substitutions contribute to the epitope of mAb 2D12 or act as modifiers of the underlying structure of the proteins and facilitate the change in the neutralization phenotype between the WT and vaccine strains of YFV.

mAb 2D12 neutralized all vaccine strains of YFV (17D and FNV) to a much lower degree than their WT parent strains. The amino acid differences between the strains likely alter the epitope for mAb 2D12 such that it can neutralize WT strains of YFV and can no longer effectively neutralize the vaccine strains. The six FNV viruses utilized in this study differ by too many residues to disentangle the LNI results to determine which substitutions have the highest effect on mAb 2D12 neutralization. However, the three 17D substrain viruses and all six FNV viruses differ from both Asibi ic virus and FVV at two positions: M-36 and E-331. These residues should be utilized in future studies to determine their individual effects on neutralization.

Other possibilities for the differences in neutralization also need to be considered. In addition to the amino acid differences between Asibi, 17D, FVV, and FNV viruses, at least two other factors may contribute to the neutralization phenotype differences. The first is that varying control infectivity titers of the viruses may lead to varying degrees of neutralization. To achieve accurate titration after neutralization, it is undesirable to use viruses with low titers. Therefore, the highest control infectivity titers possible were used in these assays to optimize the neutralization results. The samples of 17D-204 and Asibi viruses utilized for these experiments had a similar range of control titers but very different LNI values (Figure 4a). Similarly, no discernible pattern was seen between the control titers and LNI values for FVV and FNV samples (Figure 4b). Thus, there is no evidence that control infectivity titers influence the LNI in these experiments.

Asibi and FVV have previously been found to have higher genetic diversity than 17D and FNV [11,18]. This reduced genetic diversity is hypothesized to contribute to the attenuation of the 17D vaccine and FNV and could impact other viral features, such as structural epitopes. It has also been shown that structural proteins, as well as NS proteins, can affect viral genetic diversity. Exchanges of the prM and E genes between 17D and Asibi viruses showed intermediate genetic diversity between 17D and Asibi [20]. Further, some E structural epitopes differ between these WT and vaccine strain viruses, and could be important to our understanding of YFV vaccine attenuation and immunogenicity [4,21]. At the very least, mAb 2D12 can be used as a tool to distinguish WT virulent strains of YFV from live attenuated vaccine viruses.

To determine if there is a correlation between viral population genetic diversity and mAb 2D12 neutralization, the viruses were sequenced using NGS. It was possible that viruses with higher genetic diversity would be more prone to antibody neutralization escape, because higher variability may result in a higher probability of mutations at the binding site for the antibody. However, this was determined not to be the case. NGS analysis showed each strain had a unique level of viral population genetic diversity, as measured by Shannon entropy and the percentage of SNVs in a viral population (Supplemental Tables S6 and S7). Some were significantly more diverse than Asibi ic virus, some were less diverse, and some had approximately the same level of genetic diversity. However, none of these values trended with their respective LNI results, indicating that viral population genetic diversity did not significantly impact the neutralization of these viruses by mAb 2D12.

At present, the mechanism of neutralization by mAb 2D12 is unclear. It is unknown whether the mAb neutralizes the virus at the cell surface or within endosomes by preventing virus-to-cell fusion. One possible explanation for the difference in neutralization between WT and vaccine strains of YFV is that it has been previously reported that 17D and Asibi viruses enter host cells through distinct pathways [22]. Asibi enters the cell through classic clathrin-mediated endocytosis while 17D enters through a clathrin-independent pathway. It is currently unknown which pathways are utilized by FNV and FVV. Future studies should investigate the neutralization and cell entry mechanisms of FVV and FNV to elucidate any other commonalities between vaccine strains and WT strains of YFV and whether these correlate with mAb neutralization activity.

There are no reports on the characterization of epitopes for mAb 2D12 and other group C antibodies. mAbs 2E10 and 2C9 in antibody group B are known to interact with the YFV E protein in domain II, specifically with residues E-71 and E-125 [1]. Unlike group B antibodies that neutralize both vaccine strain 17D and WT strain Asibi YFV, the group C antibodies (including mAb 2D12) neutralize only Asibi. Competitive binding assays showed that mAb 2D12 and other group C antibodies do not compete for binding with antibodies from group B [4]. This evidence suggests that the epitope for mAb 2D12—and likely the epitopes of other group C mAbs—is distinct from that recognized by group B antibodies and is an as-of-yet undiscovered YF type-specific antigenic site. Additionally, the literature regarding YFV virion and protein structures is extremely limited. Monomeric structures of the E protein have been published, but the E protein exists as a dimer in nature. There is also a lack of high-resolution images of YFV 17D virions.

The limitations of this study include the aforementioned lack of knowledge of the mAb 2D12 neutralization mechanism and the lack of epitope characterization. Additionally, larger sample sizes would increase the power of the statistical analyses. Future studies can examine how changing single or multiple amino acids in the wild-type and vaccine strains of YFV affects neutralization by mAb 2D12. However, much like the limitations of this current study, there are 12 factorial (or 479 million) possible amino acid combinations that could be tested to determine their impact on YFV structure, immunogenicity, and, ultimately, determinants of vaccine efficacy. This includes analysis not only of linear epitopes but of the impact of amino acid changes on structural epitopes.

In conclusion, this study confirmed the YFV WT neutralization by mAb 2D12 and discovered that not only 17D but also FNV strains have at least a 100-fold lower level of neutralization by the LNI when compared to the wild-type strains (LNI of 2.96 vs. 1.01).

## Figures and Tables

**Table 1:** LNI results for Asibi and 17D-204 viruses in comparison to the original Schlesinger et al. 1983 LNI results [1]. Significance and *p*-values to compare Asibi and 17D-204 were calculated using Welch’s corrected *t*-test with unequal variances. The 17D-204 ic and 17D 2AR variants were also compared with Welch’s corrected *t*-test. n: number of samples; NA: not applicable; ns: not significant; **** *p*-value < 0.0001.

Virus	n	LNI	Standard Error	Significance	*p*-Value	Historically Published LNI [1]
Asibi	25	3.45	0.19	ns	NA	4.3
17D-204	31	0.05	0.06	****	<0.0001	<1.3

## Data Availability

Data and materials supporting the findings of this study are available through the NCBI Nucleotide. The data for full viral genome sequences have been deposited under the GenBank accession numbers: PX648516, PX660172-PX660175, and PX766920-PX766933. Next-generation sequencing data are available through NCBI’s Sequence Read Archive (SRA) under BioProject PRJNA1401925. SRA accession numbers are SRR36990668-SRR36990686.

## Supplementary Materials

The following supporting information can be downloaded at: https://www.mdpi.com/article/10.3390/vaccines14050430/s1, Supplemental Table S1: Amino acid differences in prM/M and E between Asibi ic virus, 17D ic, FNV isolates, and FVV. The position refers to the amino acid residue number within each protein and the amino acid is the same as in Asibi ic. Supplemental Table S2: Amino acid substitutions between Asibi and 17D substrain viruses. Amino acids are noted in single-letter code. The rightmost column indicates whether that particular amino acid substitution is present in all substrains of 17D virus. ^: glycosylation sites. Supplemental Table S3: LNI results for the 17D substrains. Welch’s one-way ANOVA for unequal variances and Dunnett’s multiple comparison tests were utilized for statistics. All samples were compared to the control sample 17D-204 ic. n: number of experimental replicates; NA: not applicable; ns: not significant; **** *p*-value < 0.0001. Supplemental Table S4: LNI results for the Asibi non-ic-derived virus. Welch’s one-way ANOVA for unequal variances and Dunnett’s multiple comparison tests were utilized for statistics. All samples were compared to the control sample Asibi ic. n: number of experimental replicates; NA: not applicable; ns: not significant; **** *p*-value < 0.0001. Supplemental Table S5: LNI results for the FNV strains after exposure to mAb 2D12. Welch’s one-way ANOVA for unequal variances and Dunnett’s multiple comparison tests were utilized for statistics. All samples were compared to the control sample Asibi ic, 17D ic, or FVV, as noted. n: number of experimental replicates; ns: not significant; NA: not applicable; * *p*-value < 0.05; ** *p*-value < 0.01; *** *p*-value < 0.001; **** *p*-value < 0.0001. Supplemental Table S6: Next-generation sequencing data of viruses harvested, including average depth of coverage obtained for each nucleotide position, average Shannon entropy of the entire viral genome, and standard deviation of that Shannon entropy. Virus names that are repeated are different samples of the same virus, with the same genomic sequence. Statistical significance of the mean Shannon entropy was calculated using one-way ANOVA with Dunnett’s multiple comparisons test with Asibi ic or 17D ic as the control group, as noted. All viruses were down-sampled to a depth of coverage of 1091 for analyses unless otherwise noted. NA: not applicable; ¶: NGS sample not down-sampled due to low depth of coverage; ns: not significant; * *p*-value < 0.05; ** *p*-value < 0.01; *** *p*-value < 0.001; **** *p*-value < 0.0001. Supplemental Table S7: The total number of SNVs for each virus sample was separated into those above 1%, 5%, and 10%. Averages do not include viral samples that were not down-sampled during NGS analysis.

## Abbreviations

The following abbreviations are used in this manuscript:

YFV	Yellow fever virus
WT	Wild type
LNI	Log neutralization index
mAb	Monoclonal antibody

## References

[1] Schlesinger J.J., Brandris M.W., Monath T.P. (1983). Monoclonal antibodies distinguish between wild and vaccine strains of yellow fever virus by neutralization, hemagglutination inhibition, and immune precipitation of the virus envelope protein. Virology.

[2] Pierson T.C., Diamond M.S. (2008). Molecular mechanisms of antibody-mediated neutralisation of flavivirus infection. Expert Rev. Mol. Med..

[3] Sil B.K., Dunster L.M., Ledger T.N., Wills M.R., Minor P.D., Barrett A.D. (1992). Identification of envelope protein epitopes that are important in the attenuation process of wild-type yellow fever virus. J. Virol..

[4] Schlesinger J.J., Walsh E.E., Brandriss M.W. (1984). Analysis of 17D Yellow Fever Virus Envelope Protein Epitopes Using Monoclonal Antibodies. J. Gen. Virol..

[5] Theiler M., Smith H.H. (1937). The use of yellow fever virus modified by in vitro cultivation for human immunization. J. Exp. Med..

[6] Theiler M., Smith H.H. (1937). The Effect of Prolonged Cultivation In Vitro Upon the Pathogenicity of Yellow Fever Virus. J. Exp. Med..

[7] Staples J.E., Barrett A.D.T., Wilder-Smith A., Hombach J. (2020). Review of data and knowledge gaps regarding yellow fever vaccine-induced immunity and duration of protection. npj Vaccines.

[8] Lloyd W., Penna H.A. (1933). Studies on the pathogenesis of neurotropic yellow fever virus in Macacus rhesus. Am. J. Trop. Med. Hyg..

[9] Davis N.C., Lloyd W., Frobisher M. (1932). The transmission of neurotropic yellow fever virus by stegomyia mosquitoes. J. Exp. Med..

[10] Smithburn K.C., Durieux C., Koerber R., Penna H.A., Dick G.W.A., Courtois G., Souza Manso C.d., Stuart G., Bonnel P.H. (1956). Yellow Fever Vaccination.

[11] Beck A.S., Wood T.G., Widen S.G., Thompson J.K., Barrett A.D.T. (2018). Analysis By Deep Sequencing of Discontinued Neurotropic Yellow Fever Vaccine Strains. Sci. Rep..

[12] Mason R.A., Tauraso N.M., Spertzel R.O., Ginn R.K. (1973). Yellow Fever Vaccine: Direct Challenge of Monkeys Given Graded Doses of 17D Vaccine. Appl. Microbiol..

[13] Monath T.P., McCarthy K., Bedford P., Johnson C.T., Nichols R., Yoksan S., Marchesani R., Knauber M., Wells K.H., Arroyo J. (2002). Clinical proof of principle for ChimeriVax^TM^: Recombinant live, attenuated vaccines against flavivirus infections. Vaccine.

[14] Bredenbeek P.J., Kooi E.A., Lindenbach B., Huijkman N., Rice C.M., Spaan W.J.M. (2003). A stable full-length yellow fever virus cDNA clone and the role of conserved RNA elements in flavivirus replication. J. Gen. Virol..

[15] McElroy K.L., Tsetsarkin K.A., Vanlandingham D.L., Higgs S. (2005). Characterization of an infectious clone of the wild-type yellow fever virus Asibi strain that is able to infect and disseminate in mosquitoes. J. Gen. Virol..

[16] Davis E.H., Beck A.S., Strother A.E., Thompson J.K., Widen S.G., Higgs S., Wood T.G., Barrett A.D.T. (2019). Attenuation of Live-Attenuated Yellow Fever 17D Vaccine Virus Is Localized to a High-Fidelity Replication Complex. mBio.

[17] Davis E.H., Thompson J.K., Widen S.G., Barrett A.D.T. (2021). Genome Characterization of Yellow Fever Virus Wild-Type Strain Asibi, Parent to Live-Attenuated 17D Vaccine, from Three Different Sources. Viruses.

[18] Beck A., Tesh R.B., Wood T.G., Widen S.G., Ryman K.D., Barrett A.D.T. (2014). Comparison of the Live Attenuated Yellow Fever Vaccine 17D-204 Strain to Its Virulent Parental Strain Asibi by Deep Sequencing. J. Infect. Dis..

[19] Nishijima N., Marusawa H., Ueda Y., Takahashi K., Nasu A., Osaki Y., Kou T., Yazumi S., Fujiwara T., Tsuchiya S. (2012). Dynamics of Hepatitis B Virus Quasispecies in Association with Nucleos(t)ide Analogue Treatment Determined by Ultra-Deep Sequencing. PLoS ONE.

[20] Collins N.D., Beck A.S., Widen S.G., Wood T.G., Higgs S., Barrett A.D.T. (2018). Structural and Nonstructural Genes Contribute to the Genetic Diversity of RNA Viruses. mBio.

[21] Cammack N., Gould E.A. (1986). Topographical analysis of epitope relationships on the envelope glycoprotein of yellow fever 17D vaccine and the wild type Asibi parent virus. Virology.

[22] Fernandez-Garcia M.D., Meertens L., Chazal M., Hafirassou M.L., Dejarnac O., Zamborlini A., Despres P., Sauvonnet N., Arenzana-Seisdedos F., Jouvenet N. (2016). Vaccine and Wild-Type Strains of Yellow Fever Virus Engage Distinct Entry Mechanisms and Differentially Stimulate Antiviral Immune Responses. mBio.

