# Supplementary material for: The Yellow Fever Virus Type-Specific Epitope Recognized by Monoclonal Antibody 2D12 Neutralizes Wild Type, but Not Live Attenuated 17D or French Neurotropic Vaccine Strains
Source: Vaccines (Basel). 2026 May 12;14(5):430. doi: 10.3390/vaccines14050430 (PMC13211704; doi:10.3390/vaccines14050430)
Supplement: Supplementary file 1 [file vaccines-14-00430-s001.zip › vaccines-4185878-supplementary.pdf]

Supplemental Table S1. Amino acid differences in prM/M and E between Asibi ic virus, 17D ic, FNV isolates, and FVV. The position refers to the amino acid residue number within each protein. - : amino acid is the same as in Asibi ic.

**Position**	**Asibi ic**	**17D ic**	**FNV-Yale**	**FNV-IP**	**FNV-FC**	**FNV-NT**	**FNV-281**	**FNV-Dakar**	**FVV**
prM-4	V	-	-	-	-	-	-	M	-
prM-25	V	-	M	M	M	I	M	I	-
prM-39	Y	-	-	-	-	H	-	-	-
prM-74	G	A	-	-	-	-	-	A	-
M-36	L	F	F	F	F	F	F	F	L
E-6	I	-	-	-	-	-	-	V	-
E-7	T	-	A	A	A	-	A	A	-
E-52	G	R	-	-	-	-	-	-	-
E-54	A	-	V	V	V	V	V	V	-
E-56	A	V	-	-	-	-	-	-	-
E-71	N	-	-	-	-	-	-	D	-
E-140	R	-	-	-	-	K	-	K	-
E-141	A	-	-	-	-	V	-	-	-
E-151	N	-	-	-	-	D	-	D	-
E-153	N	-	K	K	K	-	K	K	-
E-155	D	-	-	-	-	A	-	-	-
E-156	I	-	-	-	-	T	-	-	-
E-163	A	-	-	-	-	-	-	V	-
E-170	A	V	-	-	-	-	-	-	-
E-173	T	I	-	-	-	-	-	-	-
E-178	A	-	-	-	-	-	-	V	-
E-200	K	T	-	-	-	-	-	-	T
E-249	N	-	D	D	D	D	D	D	-
E-299	M	I	-	-	-	-	-	-	-
E-305	S	F	-	-	-	-	-	-	-
E-325	P	S	-	-	-	-	-	-	-
E-331	K	R	R	R	R	R	R	R	-
E-378	V	-	-	-	-	-	-	I	-
E-380	T	R	-	-	-	-	-	-	-
E-407	A	V	-	-	-	V	-	-	-
E-416	A	T	-	-	-	-	-	-	-
E-457	M	-	I	I	I	-	I	-	-

Supplemental Table S2. Amino acid substitutions between Asibi and 17D substrain viruses. Amino acids noted in single-letter code. The rightmost column indicates whether that particular amino acid substitution is present in all substrains of 17D virus. ^: glycosylation sites.

**Position**	**Asibi ic**	**17D-204 ic**	**17DD**	**17D-213**	**Common to all 17D vaccine substrains**
M-36	L	F	F	F	Yes
E-52	G	R	R	R	Yes
E-56	A	V	A	V	No
E-153^	N	T	N	T	No
E-155^	D	D	S	D	No
E-170	A	V	V	V	Yes
E-173	T	I	I	I	Yes
E-200	K	T	T	T	Yes
E-299	M	I	I	I	Yes
E-305	S	F	F	F	Yes
E-325	P	S	P	S	No
E-331	K	R	R	R	Yes
E-380	T	R	R	R	Yes
E-407	A	V	V	V	Yes
E-416	A	T	A	T	No

Supplemental Table S3. LNI results for the 17D Substrains. Welch's One-way ANOVA for unequal variances and Dunnett's multiple comparison tests were utilized for statistics. All samples were compared to the control sample 17D-204 ic. n: number of experimental replicates; NA: not applicable; ns: not significant; ****p-value<0.0001.

**Virus**	**n**	**LNI**	**Standard Error**	**Significance**	**p-value**
17D-204 ic	31	0.05	0.06	NA	NA
17D-204 YF-VAX®	3	0.31	0.24	ns	0.76
17DD Brazil Bio-Manguinhos, FIOCRUZ	6	0.31	0.21	ns	0.67
17-213 Russia Chumakov Institute	3	0.24	0.09	ns	0.41
Asibi ic	25	3.45	0.19	****	<0.0001

Supplemental Table S4. LNI results for the Asibi non-ic-derived virus. Welch's One-way ANOVA for unequal variances and Dunnett's multiple comparison tests were utilized for statistics. All samples were compared to the control sample Asibi ic. n: number of experimental replicates; NA: not applicable; ns: not significant; **** p-value<0.0001.

Virus	N	LNI	Standard Error	Significance	p-value
Asibi ic	25	3.45	0.19	NA	NA
Asibi Yale	3	3.80	0.21	ns	0.4239
17D-204 ic	31	0.05	0.06	****	<0.0001

Supplemental Table S5. LNI results for the FNV strains after exposure to mAb 2D12. Welch's One-way ANOVA for unequal variances and Dunnett's multiple comparison tests were utilized for statistics. All samples were compared to the control sample Asibi ic, 17D ic, or FVV, as noted. n: number of experimental replicates; ns: not significant; NA: not applicable; *p-value<0.05; **p-value<0.01; ***p-value<0.001; ****p-value<0.0001.

**Virus**	**n**	**LNI**	**Standard Error**	**Significance compared to Asibi ic**	**p-value compared to Asibi ic**	**Significance compared to 17D ic**	**p-value compared to 17D ic**	**Significance compared to FVV**	**p-value compared to FVV**
**Asibi ic**	**25**	**3.45**	**0.19**	**NA**	**NA**	****	**<0.0001**	**ns**	**0.77**
FVV	4	2.96	0.3164	ns	0.77	*	0.012	NA	NA
FNV-FC	3	1.01	0.21	***	0.0009	ns	0.16	*	0.012
FNV-Yale	5	0.85	0.36	**	0.0043	ns	0.38	*	0.019
FNV-281	6	0.81	0.33	***	0.0005	ns	0.33	**	0.0096
FNV-NT	3	0.77	0.60	ns	0.17	ns	0.84	ns	0.19
FNV-IP	3	0.74	0.08	****	<0.0001	*	0.01	*	0.027
FNV-Dakar	5	0.54	0.18	****	<0.0001	ns	0.24	**	0.0064
**17D ic**	**31**	**0.05**	**0.06**	****	**<0.0001**	**NA**	**NA**	*	**0.012**

Supplemental Table S6. Next Generation Sequencing data of viruses harvested, including average depth of coverage obtained for each nucleotide position, average Shannon entropy of the entire viral genome, and standard deviation of that Shannon entropy. Virus names that are repeated are different samples of the same virus, with the same genomic sequence. Statistical significance of the mean Shannon entropy was calculated using one-way ANOVA with Dunnett's multiple comparisons test with Asibi ic or 17D ic as the control group, as noted. All viruses were down-sampled to a depth of coverage of 1091 for analyses unless otherwise noted. NA: not applicable; ¶: NGS sample not down-sampled due to low depth of coverage; ns: not significant; *p-value<0.05; **p-value<0.01; ***p-value<0.001; ****p-value<0.0001.

Virus	Depth of Coverage	Shannon Entropy	Standard Deviation of Shannon Entropy	Significance compared to Asibi ic	p-value compared to Asibi ic	Significance compared to 17D ic	p-value compared to 17D ic
FVV	3003	0.00274	0.0117	**	0.0049	ns	0.99
**17D ic**	**3068**	**0.00278**	**0.00646**	****	**<0.0001**	**-**	**-**
FNV-NT	4180	0.00282	0.0129	*	0.014	ns	0.99
FNV-IP	997.8¶	0.00350	0.0158	ns	0.99	ns	0.075
FNV-NT	9067	0.00360	0.00825	ns	0.99	*	0.027
FNV-NT	1178	0.00366	0.0137	ns	0.99	*	0.014
**Asibi ic**	**4910**	**0.00369**	**0.01178**	**-**	**-**	**	**0.0083**
FNV-NT	6947	0.00388	0.00854	ns	0.99	***	0.0006
FNV-FC	2870	0.00390	0.0103	ns	0.99	***	0.0004
FNV-Yale	11452	0.00423	0.0249	ns	0.35	****	<0.0001
FNV-FC	6070	0.00434	0.0265	ns	0.15	****	<0.0001
FNV-Dakar	1659	0.00436	0.0219	ns	0.13	****	<0.0001
FNV-FC	3977	0.00451	0.0268	*	0.028	****	<0.0001
FNV-IP	5861	0.00458	0.0155	*	0.012	****	<0.0001
FNV-Yale	3385	0.00478	0.0250	***	0.0007	****	<0.0001
FNV-281	4585	0.00481	0.0264	***	0.0004	****	<0.0001
FNV-281	3736	0.00482	0.0264	***	0.0004	****	<0.0001
FNV-Dakar	1489	0.00499	0.0245	****	<0.0001	****	<0.0001
FNV 281	11192	0.00555	0.0254	****	<0.0001	****	<0.0001

Supplemental Table S7. The total number of SNVs for each virus sample was separated into those above 1%, 5%, and 10%. Averages do not include viral samples that were not down-sampled during NGS analysis.

**SNV Percentage**	**FVV**	**FNV-Yale**	**FNV-IP**	**FNV-FC**	**FNV-NT**	**FNV-281**	**FNV-Dakar**	**Asibi ic**	**17D ic**	**17D-204**	**17DD**	**17D-213**
**>1%**	6	45 (±4.24)	44	34.33 (±25.40)	16.25 (±4.35)	79.33 (±1.15)	55 (±16.97)	6.5 (±4.54)	3.09 (±2.63)	14	19.5 (±12.0)	37
**>5%**	4	19 (±0)	13	23.33 (±17.79)	4 (±2.31)	33 (±1)	16 (±2.83)	1.25 (±1.39)	0.27 (±0.90)	1	8.5 (±6.4)	2
**>10%**	4	18.5 (±0.71)	5	15.33 (±10.69)	2.25 (±2.63)	18 (±2)	13 (±1.41)	0.50 (±0.76)	0 (±0)	0	5.5 (±6.4)	0
